# PathFinder: mining signal transduction pathway segments from protein-protein interaction networks

**DOI:** 10.1186/1471-2105-8-335

**Published:** 2007-09-13

**Authors:** Gurkan Bebek, Jiong Yang

**Affiliations:** 1 EECS Department, Case Western Reserve University, Cleveland, OH 44106, USA

## Abstract

**Background:**

A Signal transduction pathway is the chain of processes by which a cell converts an extracellular signal into a response. In most unicellular organisms, the number of signal transduction pathways influences the number of ways the cell can react and respond to the environment. Discovering signal transduction pathways is an arduous problem, even with the use of systematic genomic, proteomic and metabolomic technologies. These techniques lead to an enormous amount of data and how to interpret and process this data becomes a challenging computational problem.

**Results:**

In this study we present a new framework for identifying signaling pathways in protein-protein interaction networks. Our goal is to find biologically significant pathway segments in a given interaction network. Currently, protein-protein interaction data has excessive amount of noise, e.g., false positive and false negative interactions. First, we eliminate false positives in the protein-protein interaction network by integrating the network with microarray expression profiles, protein subcellular localization and sequence information. In addition, protein families are used to repair false negative interactions. Then the characteristics of known signal transduction pathways and their functional annotations are extracted in the form of association rules.

**Conclusion:**

Given a pair of starting and ending proteins, our methodology returns candidate pathway segments between these two proteins with possible missing links (recovered false negatives). In our study, *S. cerevisiae *(yeast) data is used to demonstrate the effectiveness of our method.

## Background

The completion of various genome sequencing efforts has inspired many other studies to discover functionality of the genes in these genomes. The relationships among proteins determine their physical and temporal organization within the cell. By understanding these associations, we improve our understanding of cellular function and identify unknown proteins and their functions. Techniques such as the yeast two-hybrid (Y2H) system [1-6] or affinity purification followed by mass spectrometry [7,8] have been developed to uncover physical interactions between proteins. The application of such techniques to different organisms such as yeast, bacteria, fly, worm and human has revealed the interaction maps of these organisms. These maps drew a significant amount of attention and raised many questions.

Signaling pathways are chains of interacting proteins, in which proteins interact with each other to enable or disable their partners towards a biologically identified end-result [9]. These pathways transmit stimuli from the outside of the cell to transcription factors, which in turn regulate gene expression. The number of these processes shows how many ways the organism can react and respond to its environment. Discovery of signaling pathways is an important task for domain biologists. By finding new pathways, domain scientists can gain new insights on how a cell functions.

Traditionally, molecular components of signaling networks are discovered by gene knockout experiments and epistasis analysis [10]. In a knockout experiment, an organism is engineered to lack the expression and activity of one or more genes. Knockout experiments are often used to determine the functional role of a specific gene in the organism by studying the defects caused by the resulting mutation. Epistasis, the interaction between two or more genes to control a single phenotype, was introduced in 1909 by Bateson [10]. If a mutation in one gene masks the effects of a mutation in a second gene, then the first gene is said to be epistatic to the second.

Although detailed descriptions of specific linear signaling pathways are identified by using such methods, complex networks of interacting signaling pathways remain undiscovered. In summary, discovering signaling pathways appears to be a time-consuming and expensive process.

Signaling pathways have been an active research area in recent history. There are many studies in which signaling pathways were modeled using various approaches. Previously, signaling pathways were modeled through modular kinetic simulations of biochemical networks [11] and detailed integration of biochemical properties of the pathways [12]. In another study, Bayesian Networks were applied to multi-variable cell data to infer signaling pathways [13]. Correlating cancer based mRNA expression levels, autocrine receptor signaling loops were also discovered [14]. Another approach to model cellular pathways was developed based on perturbations of critical pathway components [15]. These were analyzed using DNA microarrays, quantitative proteomics, and databases of known physical interactions.

Protein-protein interaction networks (PPINs) were suggested for generating regulatory networks and cellular modeling as well [16]. Previously, they were used to predict metabolic pathways [17]. Microarray expression profiles and PPINs were also used together to form protein clusters and signaling pathways were reconstructed [18]. Similar integration was also used to order known signaling pathway components [19]. More recently, a methodology in which searching for signaling pathways is carried out on a weighted network was presented [20]. Another tool developed for searching homologous pathways queries in a given PPIN is QPath [21]. While searching for homologous structures, QPath also considers insertions and deletions of proteins in the identified pathways. Although QPath is yet another search tool, its functionality is limited by the requirement that it uses known pathways to search for homologous pathways. In this paper, we aim to efficiently find biologically meaningful (and interesting) paths. Protein-protein interactions (PPIs) of an organism can be summarized in a *graph *(network) in which each *node *represents a protein and each (undirected) *edge *represents an interaction. A graph including *all *proteins in an organism and all possible interactions between these proteins is called the *protein-protein interaction *network of that organism. Given two proteins from this network, our goal is to discover signaling pathway segments connecting these two proteins.

However, there are many challenges to this problem. First, it is very well known that, high throughput interaction discovery methods can generate false negatives (report a true interaction as not existing) or predict false positives (predict a relationship between two unrelated proteins). Although earlier studies tried to incorporate other data sources to resolve these problems, in the end, the results presented were sparse and the methodologies were not focused enough to correlate PPINs with signaling pathways. Secondly, different proteins may have similar biological functions. In other words, different pathways might have different proteins with the same functionality. Therefore, it could be troublesome using interactions in one (known) pathway to infer interactions in another (unknown) pathway.

To address the above challenges, we present a new systematic method, namely *PathFinder*, for this identification problem. In this framework, we first collect functional annotations of known pathway proteins. We map proteins to their functional annotations to capture underlying characteristics of the known pathways, since different proteins in the same annotation family may have the same biological function. These characteristics are then used to search for possible (unknown) pathway segments. Association rule mining, a procedure to collect data attributes that are statistically related in the underlying data, is used to discover patterns from pathways.

By collecting the underlying functional patterns of signaling pathways, we build a library of templates. These rules are then used to evaluate the candidate pathway segments for possible occurrences of these rules. Therefore, by extracting associations of the proteins in pathways, one can easily identify whether proteins in a given sequence are associated with each other in a similar way or not. We use the level-wise search algorithm based on the Apriori Property, an efficient association rule mining algorithm to acquire association rules for signaling pathways [22].

Next, we create a PPIN from the acquired PPIs. Techniques like the two-hybrid system or affinity purification have been widely applied to uncover physical interactions between proteins. However, these experiments identify only a small fraction of the total PPIN [23-28]. We assign reliability scores to each PPI by integrating microarray expression levels, network topology, and protein subcellular localization data and then eliminate the low-scoring interactions Gene expression profiles are integrated into our methodology to obtain more accurate information on PPIs. If a PPI is also identified as a signaling pathway interaction, then the genes producing the associated proteins should follow a similar or contrasting (activation or inhibition) level of expression. Previously, similar approaches were used to discover signaling pathways from available gene expression profiles of *S. cerevisiae *[14,18,19]. Protein subcellular localization data helps eliminating interactions among proteins that are not biologically significant. This integration would eliminate false positive PPIs, resulting from proteins that erroneously appear to coexist at the same location in a cell.

In this study, we also attempt to recover false negative PPIs. To identify these missing links, we utilize protein families. First, we identify the families of the proteins. In short, a protein family is a group of evolutionarily related proteins. Therefore, by grouping the proteins, we capture their possible interaction traits and can infer highly possible missing interactions. We grow the PPIN with these *inferred interaction edges *and carry out our searches on this extended network as well as the actual PPIN.

**Figure 1:**
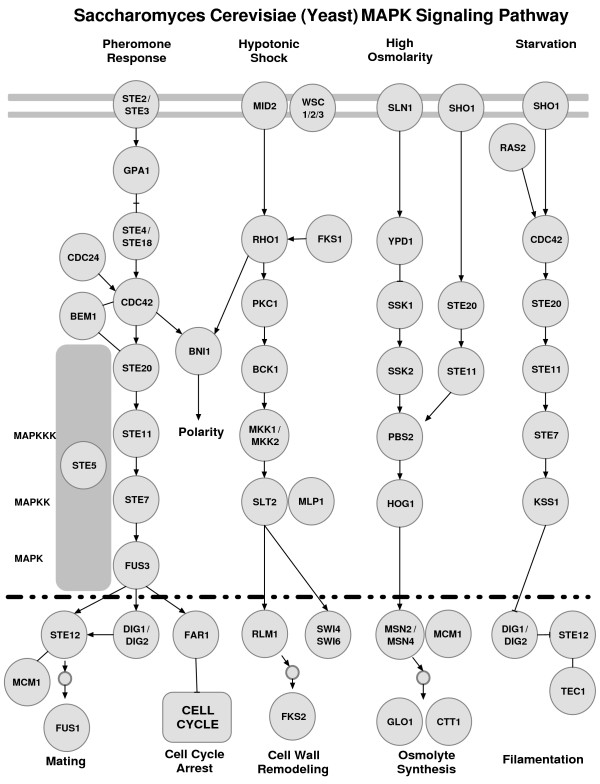
**The MAP kinase pathways**. The MAP kinase pathways downloaded from the KEGG database [30].

Finally, we query our rule set for a pair of starting and ending proteins for a pathway segment. Using this methodology, we can successfully recover previously published pathways segments accurately. In order to illustrate the success of our approach, we show that our method accurately recovers segments of well-known MAP kinase (Figure 1) pathways and performs better than previously presented studies.

### Preliminary

We now formalize the problem investigated in this paper. Let (*V*, *E*, *corr*) be the PPIN where *V *= *p*_0_, *p*_1_, *p*_2_,...*p*_*n *_is the set of all proteins, *E *= {*e *= (*p*_*i*_, *p*_*j*_)|*p*_*i*_, *p*_*j*_*εV*} is the set of interactions among these proteins and *corr *is the weight function on the edges in *E*. In this paper, *corr*(*e*) is a function of expression levels of the proteins associated with edge *e *and gives the expression correlation of these two proteins (The weight function, corr(*e*), will be discussed in detail in *Interactions with weights*).

A *pathway segment *of *length l *is an ordered list or sequence of distinct proteins in *V *such that each consecutive pair is in *E*. In other words, a pathway segment is a simple path in a PPIN. Also, each segment by definition is a part of some signaling pathways.

Given a weighted PPIN (*V*, *E*, *corr*) with proteins *V*, interactions *E *and *corr*(*e*) representing the expression correlation between connected proteins, the starting (*p*_1_) and ending nodes (*p*_l_) of a pathway segment from this network, and lower and upper bounds on the length of the pathway segment (*l*_*lower*_, *l*_*upper*_), our objective is to discover pathway segments between *p*_1 _and *p*_l _within this network with length *l *where *l*_*lower *_≤ *l *≤, *l*_*upper*_.

## Methods

Computational approaches used to discover signaling pathways were previously presented [11-15,18-21]. In each study different sets of data and methodologies were utilized for revealing pathways. In this work, we build a (search) system utilizing properties of the signaling pathways and proteins in these pathways. More precisely, we first determine the underlying relationships of proteins in pathways by capturing the characteristics of the pathways' structure. Our goal is to build a system that can answer queries on possible pathway segments using this acquired information.

The methodology described utilizes a network that embeds rich functional information and searches possible pathway segments on this network to generate a candidate pathway segment for a given query. In this study, by pooling various data sources and using efficient data mining techniques, we are essentially generating hypothesis that can be verified later by the domain scientists. Signal transduction pathways are key in understanding complex diseases such as cancer and diabetes. Hence, a tool that can return possible signaling pathway segments is quite valuable to any study that requires association of proteins functionally. In this study, to accomplish the above, we investigate signaling pathways and their functional annotations to better capture the underlying structures. We also focus on PPIs and correlate the underlying proteins with their gene expression profiles, protein families and cellular location. Here, we compare our results with two of the most promising studies in terms of wider coverage capability of the cellular mechanisms and accuracy of the methodology [18,20].

Our hypothesis is that, given association rules that capture the characteristics of signaling pathways, and a weighted PPIN that contains reliable interactions, a pathway segment should belong to a pathway if it contains at least a certain number of these rules and has an average weight of interactions above a given threshold.

We verify our hypothesis by carrying out experiments on publicly available *S. cerevisiae *data sets. The steps that are taken in this process are described in detail in the following sections. First, functional patterns of known signaling pathways are discovered by mapping pathway proteins to their functional annotations and then by mining association rules from this data set (See *Mapping proteins to functional annotations *and *Mining association rules from known pathways*). Secondly, a PPIN is created from the acquired PPI data sets by assigning reliability scores to each PPI and eliminating the low-scoring interactions (See *Gaining interaction confidence*). Next, weights are assigned to each PPI by combining microarray gene expression data of the interacting proteins (See *Interactions with weights*).

Finally, given a search query of interest – a starting and ending protein pair, bounds on the length of the pathway segment – paths connecting this protein pair are checked for the existence of any association rules. The paths that contain a significant number of rules and are above a certain co-expression level among proteins in the path are returned as results (See *Searching for pathway segments*). We also consider the scenario where a large number of false negative interactions (links) exist. The protein family information is used to recover these missing links in the PPIN. We call these recovered missing links as *inferred *links. Allowing only one *inferred *link per path, we repeat our searches to improve our results (See *Recovering false negative interactions*).

### Mapping proteins to functional annotations

In an organism, proteins may have similar biological functions, i. e. one protein may replace another protein in a pathway if they both have the same biological functionality. These proteins should share the same set of gene annotation terms. Biological annotations, e.g., Gene Ontology [29] annotations provide a basis to find functionally similar proteins. We map proteins in both known signaling pathways and protein interaction networks to their annotations. To represent relationships among proteins, we propose using functional annotations of the proteins rather than just protein names. Thus, this will strengthen the accuracy of our pathway segment prediction system.

In this study, the training data of known signaling pathways is collected from various pathways databases [30-33]. Currently, the databases for signaling pathways provide superficial images of the pathways. These databases are first converted to tuples of interacting proteins. Next, functional annotations of pathway proteins are collected and kept as functionality sets. A functional annotation scheme for mapping the proteins in an organism, such as the Gene Ontology [29] or FunCat [34] annotations can be used to describe proteins and their interactions with other macro-molecules. In this study, we chose the Gene Ontology annotations as the annotation to demonstrate the usefulness of our model. For each protein, associations between gene product and GO terms are queried from the GO Database. The ontology terms acquired are leaf nodes on the directed acyclic GO term graph. The hierarchical nature of the Gene Ontology Database *can *improve understanding of the functional assignments in further studies. However, our system is not dependent on Gene Ontology annotations, and can be easily extended or converted to other annotations if needed.

**Figure 2:**
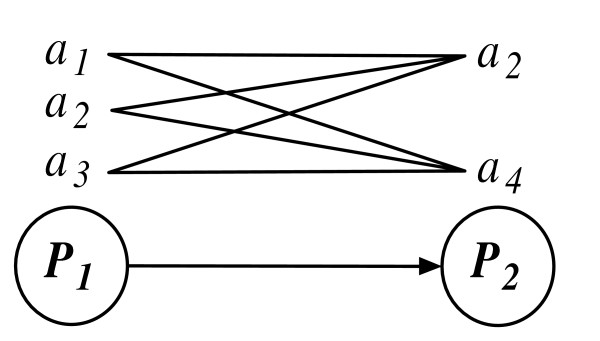
**Two interacting proteins, *P*_1 _and *P*_2_, with their respective annotation terms**. An annotation link is created by linking an annotation from the first protein's annotation set to another annotation of the second protein. Above, the tuples {(*a*_1_, *a*_2_), (*a*_1_, *a*_4_), (*a*_2_, *a*_2_), (*a*_2_, *a*_4_), (*a*_3_, *a*_2_), (*a*_3_, *a*_4_)} are shown. There are a total of 3 × 2 = 6 annotation links between *P*_1 _and *P*_2_.

Proteins in an organism may have multiple functional annotations. In this framework, for each protein an annotation set is kept. Using these annotation sets, functional relationships among proteins are established (see Figure 2). Each annotation of a protein is linked with its interacting neighbor's annotations and a network of annotation links is formed.

For instance, given proteins, (*p*_*i*_, *p*_*j*_) ∈ *E*, and their functional annotations from a known annotation scheme *G*, Fpi
 MathType@MTEF@5@5@+=feaafiart1ev1aaatCvAUfKttLearuWrP9MDH5MBPbIqV92AaeXatLxBI9gBaebbnrfifHhDYfgasaacH8akY=wiFfYdH8Gipec8Eeeu0xXdbba9frFj0=OqFfea0dXdd9vqai=hGuQ8kuc9pgc9s8qqaq=dirpe0xb9q8qiLsFr0=vr0=vr0dc8meaabaqaciaacaGaaeqabaqabeGadaaakeaacqWGgbGrdaWgaaWcbaGaemiCaa3aaSbaaWqaaiabdMgaPbqabaaaleqaaaaa@30E9@ ⊂ *G *and Fpj
 MathType@MTEF@5@5@+=feaafiart1ev1aaatCvAUfKttLearuWrP9MDH5MBPbIqV92AaeXatLxBI9gBaebbnrfifHhDYfgasaacH8akY=wiFfYdH8Gipec8Eeeu0xXdbba9frFj0=OqFfea0dXdd9vqai=hGuQ8kuc9pgc9s8qqaq=dirpe0xb9q8qiLsFr0=vr0=vr0dc8meaabaqaciaacaGaaeqabaqabeGadaaakeaacqWGgbGrdaWgaaWcbaGaemiCaa3aaSbaaWqaaiabdQgaQbqabaaaleqaaaaa@30EB@ ⊂ *G*, the Cartesian product of these sets will give all possible annotation pairs between the two proteins, i. e. *S*_*ij *_= (Fpi
 MathType@MTEF@5@5@+=feaafiart1ev1aaatCvAUfKttLearuWrP9MDH5MBPbIqV92AaeXatLxBI9gBaebbnrfifHhDYfgasaacH8akY=wiFfYdH8Gipec8Eeeu0xXdbba9frFj0=OqFfea0dXdd9vqai=hGuQ8kuc9pgc9s8qqaq=dirpe0xb9q8qiLsFr0=vr0=vr0dc8meaabaqaciaacaGaaeqabaqabeGadaaakeaacqWGgbGrdaWgaaWcbaGaemiCaa3aaSbaaWqaaiabdMgaPbqabaaaleqaaaaa@30E9@) × (Fpj
 MathType@MTEF@5@5@+=feaafiart1ev1aaatCvAUfKttLearuWrP9MDH5MBPbIqV92AaeXatLxBI9gBaebbnrfifHhDYfgasaacH8akY=wiFfYdH8Gipec8Eeeu0xXdbba9frFj0=OqFfea0dXdd9vqai=hGuQ8kuc9pgc9s8qqaq=dirpe0xb9q8qiLsFr0=vr0=vr0dc8meaabaqaciaacaGaaeqabaqabeGadaaakeaacqWGgbGrdaWgaaWcbaGaemiCaa3aaSbaaWqaaiabdQgaQbqabaaaleqaaaaa@30EB@) = {{*a*_1_, *b*_1_}, {*a*_2_, *b*_1_},...,{*a*_*n*-1_, *b*_m_}, {*a*_*n*_, *b*_*m*_}} where *a*_*r*_, *b*_*s *_∈ *G *are the annotation terms in Fpi
 MathType@MTEF@5@5@+=feaafiart1ev1aaatCvAUfKttLearuWrP9MDH5MBPbIqV92AaeXatLxBI9gBaebbnrfifHhDYfgasaacH8akY=wiFfYdH8Gipec8Eeeu0xXdbba9frFj0=OqFfea0dXdd9vqai=hGuQ8kuc9pgc9s8qqaq=dirpe0xb9q8qiLsFr0=vr0=vr0dc8meaabaqaciaacaGaaeqabaqabeGadaaakeaacqWGgbGrdaWgaaWcbaGaemiCaa3aaSbaaWqaaiabdMgaPbqabaaaleqaaaaa@30E9@ and Fpj
 MathType@MTEF@5@5@+=feaafiart1ev1aaatCvAUfKttLearuWrP9MDH5MBPbIqV92AaeXatLxBI9gBaebbnrfifHhDYfgasaacH8akY=wiFfYdH8Gipec8Eeeu0xXdbba9frFj0=OqFfea0dXdd9vqai=hGuQ8kuc9pgc9s8qqaq=dirpe0xb9q8qiLsFr0=vr0=vr0dc8meaabaqaciaacaGaaeqabaqabeGadaaakeaacqWGgbGrdaWgaaWcbaGaemiCaa3aaSbaaWqaaiabdQgaQbqabaaaleqaaaaa@30EB@ respectively. All possible combinations are examined since they represent all possible functional associations. In other words, the set *S*_*ij*_, with cardinality |Fpi|⋅|Fpj|
 MathType@MTEF@5@5@+=feaafiart1ev1aaatCvAUfKttLearuWrP9MDH5MBPbIqV92AaeXatLxBI9gBaebbnrfifHhDYfgasaacH8akY=wiFfYdH8Gipec8Eeeu0xXdbba9frFj0=OqFfea0dXdd9vqai=hGuQ8kuc9pgc9s8qqaq=dirpe0xb9q8qiLsFr0=vr0=vr0dc8meaabaqaciaacaGaaeqabaqabeGadaaakeaadaabdaqaaiabdAeagnaaBaaaleaacqWGWbaCdaWgaaadbaGaemyAaKgabeaaaSqabaaakiaawEa7caGLiWoacqGHflY1daabdaqaaiabdAeagnaaBaaaleaacqWGWbaCdaWgaaadbaGaemOAaOgabeaaaSqabaaakiaawEa7caGLiWoaaaa@3DCA@, denotes all possible annotation links between two proteins, *p*_*i*_, *p*_*j *_∈ *V*. Hence, |Fpi|⋅|Fpj|
 MathType@MTEF@5@5@+=feaafiart1ev1aaatCvAUfKttLearuWrP9MDH5MBPbIqV92AaeXatLxBI9gBaebbnrfifHhDYfgasaacH8akY=wiFfYdH8Gipec8Eeeu0xXdbba9frFj0=OqFfea0dXdd9vqai=hGuQ8kuc9pgc9s8qqaq=dirpe0xb9q8qiLsFr0=vr0=vr0dc8meaabaqaciaacaGaaeqabaqabeGadaaakeaadaabdaqaaiabdAeagnaaBaaaleaacqWGWbaCdaWgaaadbaGaemyAaKgabeaaaSqabaaakiaawEa7caGLiWoacqGHflY1daabdaqaaiabdAeagnaaBaaaleaacqWGWbaCdaWgaaadbaGaemOAaOgabeaaaSqabaaakiaawEa7caGLiWoaaaa@3DCA@ annotation tuples are generated to capture the functional relationships between *p*_*i *_and *p*_*j*_.

### Mining association rules from known pathways

Knowledge Discovery in Databases (Data Mining) is the process of discovering interesting and previously unknown information from data. There is no restriction to the type of data that can be analyzed by a data mining method. Hence, in general data mining can help extract knowledge from large amounts of data. The next step in the PathFinder framework is to capture the underlying characteristics of known signaling pathways. Proteins on a signaling cascade interact with each other enabling or disabling each other with a common functionality. Therefore, using the functional annotations instead of the proteins themselves would better capture the true relationships among proteins in known pathways.

The aim of mining association rules is to discover rules, *P *⇒ *Q*, that stand out from all other pairs in terms of significance and interest. In this work the Support-Confidence framework is used to measure significance and interest (described below). Whereas other methods can be used for finding the characteristics of the known pathways [35], association rules have returned the best results for the given problem.

Let *H *be the set of interactions in known signaling pathways (*H *⊆ *E*). Let *U *be the union of tuples generated from interacting proteins on known pathways, i.e. U=∪(pi,pj)∈HSij
 MathType@MTEF@5@5@+=feaafiart1ev1aaatCvAUfKttLearuWrP9MDH5MBPbIqV92AaeXatLxBI9gBaebbnrfifHhDYfgasaacH8akY=wiFfYdH8Gipec8Eeeu0xXdbba9frFj0=OqFfea0dXdd9vqai=hGuQ8kuc9pgc9s8qqaq=dirpe0xb9q8qiLsFr0=vr0=vr0dc8meaabaqaciaacaGaaeqabaqabeGadaaakeaacqWGvbqvcqGH9aqpdaWeqbqaaiabdofatnaaBaaaleaacqWGPbqAcqWGQbGAaeqaaaqaaiabcIcaOiabdchaWnaaBaaameaacqWGPbqAaeqaaSGaeiilaWIaemiCaa3aaSbaaWqaaiabdQgaQbqabaWccqGGPaqkcqGHiiIZcqWGibasaeqaniablQIivbaaaa@3FCA@, which is also equal to *U *= {(*a*_*r*_, *b*_*s*_)|(*p*_*i*_, *p*_*j*_) ∈ *H*, *a*_*r *_∈ Fpi
 MathType@MTEF@5@5@+=feaafiart1ev1aaatCvAUfKttLearuWrP9MDH5MBPbIqV92AaeXatLxBI9gBaebbnrfifHhDYfgasaacH8akY=wiFfYdH8Gipec8Eeeu0xXdbba9frFj0=OqFfea0dXdd9vqai=hGuQ8kuc9pgc9s8qqaq=dirpe0xb9q8qiLsFr0=vr0=vr0dc8meaabaqaciaacaGaaeqabaqabeGadaaakeaacqWGgbGrdaWgaaWcbaGaemiCaa3aaSbaaWqaaiabdMgaPbqabaaaleqaaaaa@30E9@, *b*_*s *_∈ Fpj
 MathType@MTEF@5@5@+=feaafiart1ev1aaatCvAUfKttLearuWrP9MDH5MBPbIqV92AaeXatLxBI9gBaebbnrfifHhDYfgasaacH8akY=wiFfYdH8Gipec8Eeeu0xXdbba9frFj0=OqFfea0dXdd9vqai=hGuQ8kuc9pgc9s8qqaq=dirpe0xb9q8qiLsFr0=vr0=vr0dc8meaabaqaciaacaGaaeqabaqabeGadaaakeaacqWGgbGrdaWgaaWcbaGaemiCaa3aaSbaaWqaaiabdQgaQbqabaaaleqaaaaa@30EB@}. Although *U *consists of every possible annotation tuple in *H*, it is quite intuitive that not every tuple in *U *is meaningful. An association rule is an annotation tuple (a single annotation edge) that represents a highly likely functional relationship between two proteins in the context of signaling pathways. The level-wise search algorithm based on the Apriori Property [22] is used to extract association rules among all possible annotation tuples. Please note that although proteins in both known signaling pathways and protein interaction networks are mapped into annotations, the association rules are extracted only from the known signaling pathways.

More formally, let us assume that *C *is a collection of extracted annotation pairs. Given an association rule, *R *∈ *C*, this rule implies that whenever one of the annotations in *R *is *P*, probably the other should be *Q*. The fraction of relationships *R *supporting *P *with respect to all pairs is called the *support *of *P*, *support*(*P*) = |{*R *∈ *C*|*P *⊆ *R*}|/|*C*|. The *rule confidence *is defined as the percentage of relations containing *Q *in addition to *P *with regard to the overall number of relations containing *P*. In other words, rule confidence is probability *Pr*(*Q *⊆ *R*|*P *⊆ *R*).

Association rule mining is a powerful pattern discovery method that has been popular since its introduction [22]. Although general association rules have the greater power in correlating multiple items at once, given our limitation in the number of known pathways, single associations are used. The association rules acquired in this study are similar to rules that might be identified through a brute force probability distribution of these rules (i.e., keeping higher frequency rules and eliminating others). Thus, a strip-down version of the level-wise search algorithm based on the Apriori Property is employed. It is used to find associations with support and confidence values above some thresholds. This algorithm uses a "bottom up" approach. First the frequent single annotation terms are discovered. Second, frequent annotation pairs are extracted. Finally, low confidence rules are removed and only the high-confidence rules involving annotation pairs are returned [22].

First, a set of *tuples *is formed by pairing the functional annotations of two consecutive proteins on a pathway. Association rules are pairs from this set of pairs which can satisfy constraints on measures of significance and interest (these measures are determined by calculating the support and confidence of annotations, described below). Support is first used to find frequent (significant) association rules. Then confidence is used in the second step to produce high accuracy rules.

In short, an association rule is an expression *P *⇒ *Q*, where *P *and *Q *are functional annotations. The meaning of such rules is quite intuitive: if *P *is an annotation of a pathway protein, then the protein with annotation *Q *is likely to appear next to *P *in this pathway. To derive association rules, the co-occurrences of different annotations that appear with the highest frequencies are identified.

*Support *is used as a measure of the significance (importance) of an annotation. Basically this measure uses the count of pairs (also called a frequency constraint). An annotation with a support value higher than a set minimum support threshold is called a frequent annotation. A higher support value would show that the rule, or the relationship between two annotations has appeared more than a certain number of times. Therefore, a rule with high support would be a pair of annotations describing a common functional relationship on known pathways.

*Confidence *is defined as the probability of seeing a rules-implied pair (*Q*, the consequent item) given the condition that the relationship contains the first item (*P*, the antecedent item). Higher confidence values would show that an annotation rule is more accurate in guessing the likelihood of the consequent annotation given the antecedent annotation. So, if a rule containing *P *and *Q *is known to have a high confidence value, then *Q *is more likely to appear whenever annotation *P *is seen. (Interested reader please refer to [36].)

**Figure 3:**
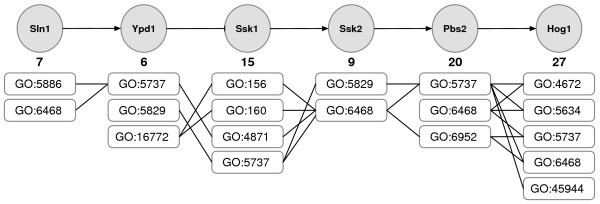
**Association rules in Sln1-Hog1 segment**. High Osmolarity Pathway Sln1-Hog1 Segment and the association rules on this pathway segment are shown. For each protein only GO [29] terms that are in an association rule are drawn. The number of GO terms each protein has are given below each protein.

In Figure 3, some of the association rules obtained are shown on the Sln1-Hog1 Signaling Pathway Segment. Originally, Hog1 has 27 and Pbs2 has 20 GO annotations. Association rule mining is a powerful tool to filter these associations such that only functionally associated rules are generated. In this example, among all possible 540 annotation pairs of Pbs2 and Hog1, only nine of them are considered as significant association rules representing the functional links between these two proteins. For instance, GO:6468 (protein amino acid phosphorylation) and GO:4672 (protein kinase activity) tuple is an excellent example of how a signal is transmitted by phosphorilating Pbs2 to trigger the kinase activity of Hog1.

By collecting the underlying functional patterns of signaling pathways, we form a library of templates. The pathway interactions generated more than 10^5 ^associations among collected functional annotations. Given that our collection of pathways interactions are less than 10^3^, we experimented on various support and confidence thresholds to generate a network of annotations that is both small in number and also can cover almost every pathway interaction link on hand. Given these requirement, we considered support threshold values between 10^-3 ^and 10^-7^, and confidence threshold values between 0.1 and 10^-5^. We then checked the size of the association rule set generated by these parameters. We observed that 2500 association rules can cover our pathways and give highly filtered annotation associations. The data used in this study generated the best results with 2582 observed association rules when the support value is 0.0001 and the confidence is 0.001.

These rules and parameters are then used to evaluate candidate pathway segments for possible occurrences of these rules. By extracting the associations of the proteins in pathways, one can easily identify whether a given sequence of proteins are interacting with each other in a similar way or not.

### Construction of a reliable and weighted protein-protein interaction network

#### Gaining interaction confidence

The PPIs identified so far have mostly been acquired by high throughput Y2H screens [1-6] or inferred from the mass spectrometry of coimmunoprecipitated protein complexes (Co-IP) [7,8]. However, analysis based on the agreement of the interaction and expression data shows that less than half of these interactions are biologically relevant [37]. Therefore, even if two proteins are observed to have an interaction, they may not be functionally related. This creates a challenge in identifying the true positive interactions that are needed for further discoveries, such as identifying the signaling pathways.

In recent years, system wide studies excluding Y2H screens and the Co-IP of protein complexes have generated a quite large number of data sets. These data sources can be integrated with known PPINs to increase the number of true positives and their reliabilities.

Many approaches [37-41] and supporting studies [42] were previously presented to improve the reliability of PPIs. By utilizing various combinations of known data sources, such as microarray expression profiles, functional annotations etc. one can assess the confidence of known PPIs.

Suthram *et al*. [43] compared these studies with respect to different quality schemes. In this study we have utilized a modified version of the Sharan *et al*. [40] method for evaluating PPI confidence values. It was observed that this methodology performs better than others in correlating functional assignments of proteins. Since our framework is based on the functional enrichment of proteins, we have based our confidence evaluation on this methodology. It was also noted that this methodology showed lower correlation with expression levels. To overcome this drawback we have also used the expression correlations by themselves.

In our logistic regression model, we incorporate four sets of variables for a given interaction set of an organism. We acquired: (1) the number of times an interaction between two proteins was observed [38,40], (2) the Pearson correlation of expression measurements for the corresponding genes, (3) the proteins' small world clustering coefficient [40], and (4) the binary (0/1) protein subcellular localization data of interacting partners [44].

Here in addition to the previously presented first three random variables [40], we also incorporate the protein subcellular localization data into the logistic model. Intuitively, this would eliminate interactions among proteins that are not biologically significant. This is very straight-forward since in most of the signaling cascades the proteins would transmit the signal from the membrane, where the signal is initiated, towards to the nucleus, where the final product is transcribed. Although proteins travel in a cell and can coexist in multiple compartments, this classification eliminates false negatives that are way off the charts. Given the four input variables, *X *= (*X*_1_, *X*_2_, *X*_3_, *X*_4_), the probability of a true interaction *Pr*(*I*_*uv*_) under the logistic distribution is given by Pr(Iuv|X)=11+exp(−β0−∑i=14βiXi)
 MathType@MTEF@5@5@+=feaafiart1ev1aaatCvAUfKttLearuWrP9MDH5MBPbIqV92AaeXatLxBI9gBaebbnrfifHhDYfgasaacH8akY=wiFfYdH8Gipec8Eeeu0xXdbba9frFj0=OqFfea0dXdd9vqai=hGuQ8kuc9pgc9s8qqaq=dirpe0xb9q8qiLsFr0=vr0=vr0dc8meaabaqaciaacaGaaeqabaqabeGadaaakeaaieGacqWFqbaucqWFYbGCcqGGOaakcqWGjbqsdaWgaaWcbaGaemyDauNaemODayhabeaakiabcYha8jabdIfayjabcMcaPiabg2da9maalaaabaGaeGymaedabaGaeGymaeJaey4kaSIaemyzauMaemiEaGNaemiCaaNaeiikaGIaeyOeI0ccciGae4NSdi2aaSbaaSqaaiabicdaWaqabaGccqGHsisldaaeWaqaaiab+j7aInaaBaaaleaacqWGPbqAaeqaaOGaemiwaG1aaSbaaSqaaiabdMgaPbqabaGccqGGPaqkaSqaaiabdMgaPjabg2da9iabigdaXaqaaiabisda0aqdcqGHris5aaaaaaa@528C@, where *β*_0_,...,*β*_4_, are parameters of the distribution. Given positive and negative training data sets, one can optimize the parameters to maximize the likelihood of a true interaction. We have acquired randomly selected 1000 PPIs from MIPS [31] interactions, an accepted gold standard as our positive data set. The negative training set was 1000 randomly selected PPIs that are not in the golden data set of MIPS [31]. This choice is backed by the high abundance of false positives in the training data sets and has been also used in previous studies [20,40]. We repeated these experiments and optimized a cut off point for the probability of a true interaction. The PPIN downloaded had about 83600 interactions among 6301 proteins [45]. We acquired a network with 14995 true interactions through this process. From this point on PPIN stands for the network of true interactions gained through this process.

#### Interactions with weights

If an interaction participates in a signaling pathway, then the genes producing the associated proteins should be co-expressed and might be co-regulated. Previously, Grigoriev *et al*. [42] showed that biologically relevant interacting proteins have high mRNA expression correlations. Since proteins used in the same signaling network must exist simultaneously at the time of their activation, the genes encoding these proteins must be transcribed at approximately the same time, and under the same environmental conditions in which the signaling network is required. This fact was used to discover signaling pathways from available gene expression profiles of *S. cerevisiae *[18] and human cancer cells [14]. The same approach was used to order signaling pathways [19]. In this study, gene expression profiles are integrated into our methodology to obtain more accurate information on PPIs.

Systematic methods for organizing gene expression data require a means of quantitatively measuring if two expression profiles are similar to each other or not. First, the values that form the expression profile for a single gene is considered as a series of coordinates.

This definition makes it easier to consider the data for a microarray experiment as a matrix. In this matrix, the genes define the rows, and experiments define the columns. One can easily use standard mathematical techniques such as the Pearson Correlation to measure the similarity of these profiles. The Pearson Correlation treats the vectors as if they are the same (unit) length, and is thus insensitive to the amplitude of changes that may be seen in the expression profiles. This results in a distance metric that can be used to measure how similar two expression vectors are.

A *weighted *PPIN is thus formed by calculating Pearson correlation coeffecient of the interacting pairs' gene expression levels. In other words, for each pair of interacting proteins *p*_*i *_and *p*_*j*_, *e *= (*p*_*i*_, *p*_*j*_) where *e *∈ *E*, *corr*(*e*) is the Pearson correlation of the interacting pairs of proteins. For expression levels of *p*_*i *_and *p*_*j *_with *n *readings, pix
 MathType@MTEF@5@5@+=feaafiart1ev1aaatCvAUfKttLearuWrP9MDH5MBPbIqV92AaeXatLxBI9gBaebbnrfifHhDYfgasaacH8akY=wiFfYdH8Gipec8Eeeu0xXdbba9frFj0=OqFfea0dXdd9vqai=hGuQ8kuc9pgc9s8qqaq=dirpe0xb9q8qiLsFr0=vr0=vr0dc8meaabaqaciaacaGaaeqabaqabeGadaaakeaacqWGWbaCdaWgaaWcbaGaemyAaK2aaSbaaWqaaiabdIha4bqabaaaleqaaaaa@314D@ and pjx
 MathType@MTEF@5@5@+=feaafiart1ev1aaatCvAUfKttLearuWrP9MDH5MBPbIqV92AaeXatLxBI9gBaebbnrfifHhDYfgasaacH8akY=wiFfYdH8Gipec8Eeeu0xXdbba9frFj0=OqFfea0dXdd9vqai=hGuQ8kuc9pgc9s8qqaq=dirpe0xb9q8qiLsFr0=vr0=vr0dc8meaabaqaciaacaGaaeqabaqabeGadaaakeaacqWGWbaCdaWgaaWcbaGaemOAaO2aaSbaaWqaaiabdIha4bqabaaaleqaaaaa@314F@ where 1 ≤ *x *≤ *n*, with means p¯i
 MathType@MTEF@5@5@+=feaafiart1ev1aaatCvAUfKttLearuWrP9MDH5MBPbIqV92AaeXatLxBI9gBaebbnrfifHhDYfgasaacH8akY=wiFfYdH8Gipec8Eeeu0xXdbba9frFj0=OqFfea0dXdd9vqai=hGuQ8kuc9pgc9s8qqaq=dirpe0xb9q8qiLsFr0=vr0=vr0dc8meaabaqaciaacaGaaeqabaqabeGadaaakeaacuWGWbaCgaqeamaaBaaaleaacqWGPbqAaeqaaaaa@2FB4@ and p¯j
 MathType@MTEF@5@5@+=feaafiart1ev1aaatCvAUfKttLearuWrP9MDH5MBPbIqV92AaeXatLxBI9gBaebbnrfifHhDYfgasaacH8akY=wiFfYdH8Gipec8Eeeu0xXdbba9frFj0=OqFfea0dXdd9vqai=hGuQ8kuc9pgc9s8qqaq=dirpe0xb9q8qiLsFr0=vr0=vr0dc8meaabaqaciaacaGaaeqabaqabeGadaaakeaacuWGWbaCgaqeamaaBaaaleaacqWGQbGAaeqaaaaa@2FB6@, and standard deviations Spi
 MathType@MTEF@5@5@+=feaafiart1ev1aaatCvAUfKttLearuWrP9MDH5MBPbIqV92AaeXatLxBI9gBaebbnrfifHhDYfgasaacH8akY=wiFfYdH8Gipec8Eeeu0xXdbba9frFj0=OqFfea0dXdd9vqai=hGuQ8kuc9pgc9s8qqaq=dirpe0xb9q8qiLsFr0=vr0=vr0dc8meaabaqaciaacaGaaeqabaqabeGadaaakeaacqWGtbWudaWgaaWcbaGaemiCaa3aaSbaaWqaaiabdMgaPbqabaaaleqaaaaa@3103@ and Spj
 MathType@MTEF@5@5@+=feaafiart1ev1aaatCvAUfKttLearuWrP9MDH5MBPbIqV92AaeXatLxBI9gBaebbnrfifHhDYfgasaacH8akY=wiFfYdH8Gipec8Eeeu0xXdbba9frFj0=OqFfea0dXdd9vqai=hGuQ8kuc9pgc9s8qqaq=dirpe0xb9q8qiLsFr0=vr0=vr0dc8meaabaqaciaacaGaaeqabaqabeGadaaakeaacqWGtbWudaWgaaWcbaGaemiCaa3aaSbaaWqaaiabdQgaQbqabaaaleqaaaaa@3105@ respectively, the Pearson correlation coefficient for *p*_*i *_and *p*_*j *_is;

corr(e)=∑1≤x≤n(pix−p¯i)(pjx−p¯j)(n−1)SpiSpj
 MathType@MTEF@5@5@+=feaafiart1ev1aaatCvAUfKttLearuWrP9MDH5MBPbIqV92AaeXatLxBI9gBaebbnrfifHhDYfgasaacH8akY=wiFfYdH8Gipec8Eeeu0xXdbba9frFj0=OqFfea0dXdd9vqai=hGuQ8kuc9pgc9s8qqaq=dirpe0xb9q8qiLsFr0=vr0=vr0dc8meaabaqaciaacaGaaeqabaqabeGadaaakeaacqWGJbWycqWGVbWBcqWGYbGCcqWGYbGCcqGGOaakcqWGLbqzcqGGPaqkcqGH9aqpdaaeqbqaamaalaaabaGaeiikaGIaemiCaa3aaSbaaSqaaiabdMgaPnaaBaaameaacqWG4baEaeqaaaWcbeaakiabgkHiTiqbdchaWzaaraWaaSbaaSqaaiabdMgaPbqabaGccqGGPaqkcqGGOaakcqWGWbaCdaWgaaWcbaGaemOAaO2aaSbaaWqaaiabdIha4bqabaaaleqaaOGaeyOeI0IafmiCaaNbaebadaWgaaWcbaGaemOAaOgabeaakiabcMcaPaqaaiabcIcaOiabd6gaUjabgkHiTiabigdaXiabcMcaPiabdofatnaaBaaaleaacqWGWbaCdaWgaaadbaGaemyAaKgabeaaaSqabaGccqWGtbWudaWgaaWcbaGaemiCaa3aaSbaaWqaaiabdQgaQbqabaaaleqaaaaaaeaacqaIXaqmcqGHKjYOcqWG4baEcqGHKjYOcqWGUbGBaeqaniabggHiLdaaaa@6211@

Notice that the correlation of two interacting proteins is the Pearson correlation of the expression levels of the genes that produce these proteins. If *g*_1 _produces *p*_*i *_and gene *g*_2 _produces *p*_*j*_, then the correlation of *p*_*i *_and *p*_*j *_is measured as the correlation of the expression level of *g*_1 _and *g*_2_. The Pearson correlation coefficient measures the strength of a linear relationship between two expression levels of gene products. The Pearson correlation coefficient would be between -1 and +1. By definition, a positive correlation is evidence of a general tendency that high levels of *g*_1 _are associated with high levels of *g*_2 _and low levels of *g*_1 _are associated with low expression levels of *g*_2_. On the other hand, a negative correlation is evidence of a general tendency that high expression values of *g*_1 _are associated with low expression levels of *g*_2 _and low expression levels of *g*_1 _are associated with high expression levels of *g*_2_. The closer the correlation is to +1 or -1, the closer to a perfect linear relationship would two genes have. In this study, the absolute value of *corr*(*e*) is used to capture inhibitory activity of genes (negative correlation) as well as activation of genes (positive correlation). In most cases, as |*corr*(*e*)| approaches to zero, there would be little or no association among the pair of genes in consideration. Usually the correlation of the expression genes provides some evidence as to whether the produced proteins are biologically related. In our study, by carrying out an experiment among the pathways we have observed for a given pathway the average |*corr*(*e*)| of consecutive proteins is higher than 0.6. This observation is made with the study of currently available data. 1089 yeast microarray experiments were downloaded from The Stanford Microarray Database [46].

### Searching for pathway segments

PathFinder is used to search for signaling pathway segments. For this task, the acquired association rules underlying the characteristics of signaling pathways are used. As per our hypothesis, if a candidate pathway segment contains characteristics similar to some known pathways, it is highly probable that these segments belong to some known or unknown pathway as well. In other words, given association rules that capture the characteristics of some known pathways, and a weighted PPIN, a pathway segment should belong to a pathway if it contains at least a certain number of these rules and the average weight of interactions is above a given threshold.

Given initial and end proteins of a pathway segment, every possible path with length between *l*_*lower *_and *l*_*upper *_forming a simple path is checked. In our experiments we observed that having 4 ≤ *l *≤ 5 returns the most positive results. This is due to the nature of the data that is used. The length of the most known (connected) pathway segments of the yeast PPI are between 4 and 5 proteins. Although an exhaustive search seems impractical, the algorithm's run time is acceptable. While other randomized approaches might skip some of the paths, our algorithm would consider all paths. Here, a modified version of *Depth-First Search, All Simple Paths Depth-First Search *is used [36].

Candidate pathway segments are then filtered through the association rules as follows. First, each pair of interacting proteins' functional annotations are acquired. The annotation pairs formed from these two sets are then checked for a match with a tuple from the association rules set. For each path, a counter is kept, and those paths with the most rules are picked (top 10% of the paths).

For each selected path, an average *absolute *expression correlation coefficient is calculated. An average absolute expression correlation coefficient is used since not only positive but also negative correlations are also taken into account in signaling pathways. This value is then compared with a threshold. This extra filtering improves the throughput of the methodology since the filtering technique picked for calculating the probabilities of true interactions showed lower correlation with expression levels [43]. Although correlation between expression levels is utilized in gaining interaction confidence to a certain level, these values are more apparent while associating the interactions in signaling paths. The threshold for the average expression correlation is determined by calculating the average absolute value for known pathways. For the data in our experiments, the average absolute threshold was 0.6. The candidate paths with average coefficients higher than the threshold are returned as the query results. An empty set is returned when every path is eliminated through these steps.

In our experiments, we have observed that by filtering the paths through the association rules, we can prune 90% of simple pathways connecting two proteins. On the other hand, using average absolute expression correlation values, 76% of paths can be eliminated. Both of these methods can filter out incorrect paths, and retain the correct pathway in the resulting set. Hence, the association rule has the greater pruning power on filtering incorrect paths. However, when two methods are combined, 95% incorrect paths are pruned on average, which is better than using only one of these two methods.

### Recovering false negative interactions

One of the biggest challenges one can face while working with PPIs is dealing with false negatives. We attempted to eliminate false positives by integrating other data sources. To our best knowledge, no previous network search methodology has addressed recovering false negatives. Here, we integrate protein families with the PPIN to predict possible false negatives.

A protein family is a group of evolutionarily related proteins. Protein families are established in large scale based on sequence similarity. Earlier, it has been also observed that, sequence-wise similar proteins share similar interaction patterns in the same organism [47]. Therefore, proteins that are in the same family should have similar interaction patterns.

We first checked every interacting protein and identified their families. The Protein Family data was downloaded from Pfam [48]. Pfam is a database of multiple alignments of protein domains or conserved protein regions. These represent some evolutionary conserved structure which has implications for the protein's function. The curated part of Pfam contains over 8957 protein families. Next, we add *inferred interaction edges *to our network. We link the proteins in our PPIN with an inferred interaction edge, if (1) they do not interact with each other in the PPIN, and (2) there exists at least one (real) interaction between the families of these two proteins. For instance, assume that the genome of our model organism has proteins *p*_1_, *p*_2_, *q*_1 _and *q*_2_. Let us also assume that *p*_1 _and *p*_2 _belong to family *A*, *q*_1 _and *q*_2 _belong to family *B*, and proteins *p*_1 _and *q*_1 _interact but *p*_2 _and *q*_2 _do not interact with each other. We link proteins *p*_2 _and *q*_2 _with an inferred link, since members of *A *and *B *has an interaction connecting *A *and *B*.

Next, we search for a pathway segment using this extended PPIN. We follow the same search methodology described above. Once again, every possible path with a length between *l*_*lower *_and *l*_*upper *_is checked. However, this time we only allow at most one inferred edge on each simple path. Since the number of edges is increased by including the inferred edges, the searches took longer, up to 20 times depending on the degree of the proteins in consideration. Nevertheless, possible missing links can be recovered. Here, to make sure the secondary edges are correctly identified, we have only considered secondary edges with at least five association rules (this is close to the average number of association rules on interacting pathway proteins).

## Results and discussion

In this study, we carried out experiments on current, publicly available *S. cerevisiae *data to prove the validity of our model. The gene expression calculations were done using the yeast microarray data downloaded from The Stanford Microarray Database [46] and signaling pathways were collected from various databases [30-33]. The PPI data was downloaded from the MIPS Comprehensive Yeast Genome Database [31] and BioGRID [45].

Our methodology was implemented and tested for MAP Kinase (MAPK) signaling pathway segments (Figure 1). First, we picked two different random proteins from a known signaling pathway. We identified the pathway and *removed *it from our training data set. After forming association rules from other known pathways, we searched for the known pathway on the weighted PPIN. For each pathway segment we looked for, we queried the PPIN for paths with length ± 1 of the original length acquired from the pathway. After conducting 25 random experiments, we had a 73% recall and 34% precision in recovering the pathway segments. The recall and precision were obtained using PathFinder on the network with inferred links. *Recall *is the percentage of the recovered edges with respect to the original pathway segment that we are looking for. So, if all edges are found in the resulting segment, we say that the recall for that experiment is 100%. These numbers are mostly affected by the incompleteness of the PPIN, i.e. an interaction existing on a pathway does not occur on the PPIN. Therefore some of the pathway segments were impossible to rediscover completely. However, all experiments returned pathway segments as a result. *Precision *is the rate of the number of edges that are truly recovered to the total number of edges in the resulting set. This rate increases when the discovered pathway segments have smaller number of supplementary links. However, considering the high false positive and false negative rates of the PPIN, the current rate we acquired is expected.

**Figure 4:**
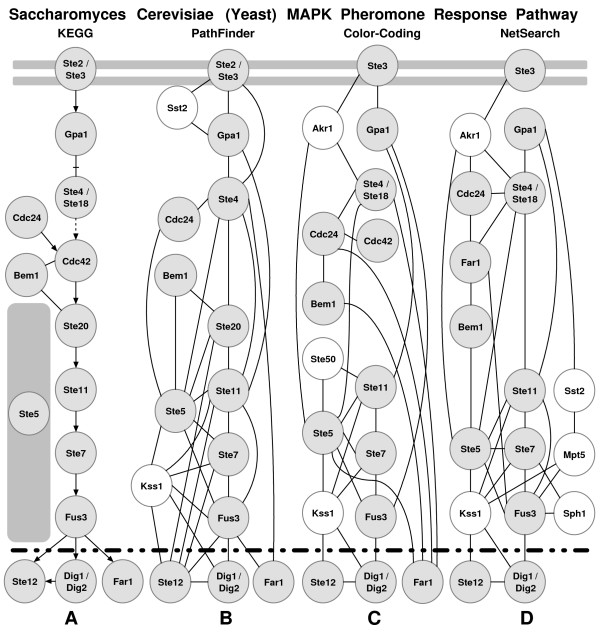
**The pheromone response signaling pathway**. (A) The main chain of the pheromone pathway downloaded from KEGG, (B) the output of our PathFinder implementation, (C) The color-coding algorithm output for the pheromone pathway [20], (D) the NetSearch program prediction for the pheromone pathway [18]. The interactions that do not exist in the PPIN are shown with dashed edges in (A). Notice that, for (B),(C) and (D) the proteins that were not on the main chain of the pathway, as shown in (A), were not colored.

Now we analyze the performance of PathFinder on some particular signaling pathways. Pheromone response and filamentous growth pathways were analyzed previously in [18,20]. We used Pathfinder to recover these specific pathways as well. The pheromone response pathway (Figure 1) triggers the yeast cell for mating by inducing polarized cell growth toward a mating partner. This pathway consists of ten cascading proteins with additional proteins assisting or binding on the sides. First, the pathway segment was excluded from the training data set. Given the yeast PPIN and the starting and ending proteins of the pheromone response pathway (Ste2-Ste12), PathFinder (without inferred links) generated the pathway shown in Figure 4B. However, note that the PPIN that is currently available is known to be incomplete. When the yeast PPIs are compared with the pheromone response pathway interactions [30], the interaction between Ste4/8 and Cdc42 does not exist in the yeast PPIN.

Given that there are missing links, PathFinder actually recovered *all *possible links that are available to us on this pathway segment. When compared with previous studies, for this particular example, PathFinder has a 78% recall and 40% precision in recovering this pathway segment (Figure 1). The color-coding algorithm output for this pathway had a 50% recall and 32% precision [20], whereas the NetSearch program prediction for the pheromone pathway had a 44% recall and 24% precision [18]. Moreover, the resulting pathway segments of PathFinder just consists of proteins from the original pathway and two additional proteins, Kss1 (a MAP Kinase) and Sst2.

Kss1 does not have a part in the pheromone signaling pathway. However, as discovered with PathFinder during this experiment and earlier by Madhani *et al *[49], by switching places with Fus3, a MAPK regulating mating, Kss1 MAPK regulates filamentation and invasion (See Figure 1). Remarkably, with similarities in kinase-dependent activation functions, Kss1 and Fus3 each have a distinct kinase-independent inhibitory function. This example shows how PathFinder, a model built on characteristics of pathways, is capable of filtering proteins according to their functions.

Sst2, a negative regulator of pheromone signaling in yeast, has a physical association with Gpa1 [50] and Ste2 [51]. At first, this additional protein on the resulting pathway looks like a false positive. However, it was noted that Sst2 *is *related to this pathway segment. Hence, our methodology may also be used to extend known signaling pathways. In this example, a network with inferred links did not improve our results.

**Figure 5:**
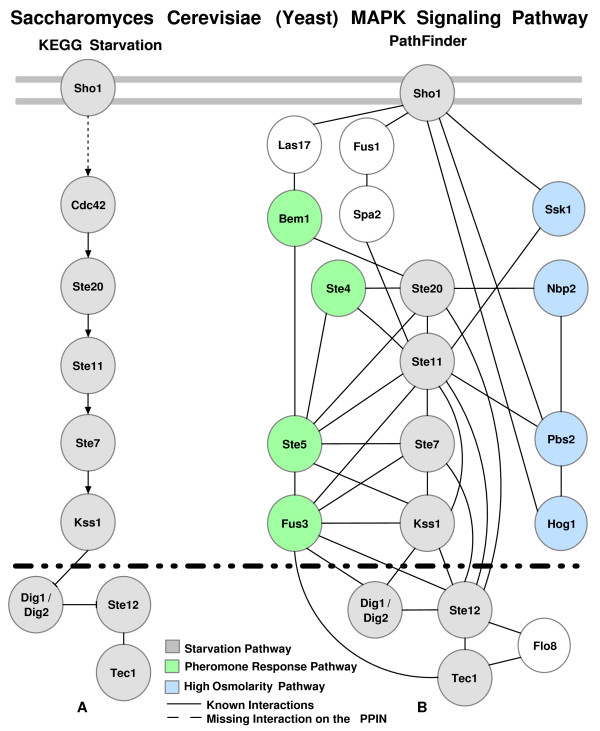
**The filamentation signaling pathway**. (A) The main chain of the filamentation pathway (KEGG Database), (B) PathFinder output for the Sho1-Tec1 pair (75% recall, 17% precision). For (A), the dashed interactions indicate that these interactions do not exists in the database. For (B), the proteins that were not on the main chain of the pathway were not colored, whereas the proteins on the main chain are colored grey. Proteins that are part of other pathways are colored with different colors.

In this second example, the filamentation MAPK pathway is searched. The filamentation MAPK pathway (Figure 5A) is activated by glucose or nitrogen starvation and results in filamentous growth. The Sho1-Tec1 protein pair is picked as the starting and ending protein pair. However, this pathway has a missing interaction in the yeast PPIN. Previously, such missing links were noted to prevent attempts to recover signaling pathways segments [18]. After searching for the pathway with our methodology (without inferred links), we acquired the pathway segment shown in Figure 5B. When the results are compared, all known interactions among the interacting proteins but Cdc42 were recovered. Additional proteins from the pheromone response and high osmolarity glycerol (HOG) MAPK pathways were also recovered. This is most likely due to shared proteins on these pathways.

In the resulting pathway segment, we have both segments from other MAPK pathways and proteins that are not mentioned in the KEGG Pathway database. Here, Flo8 interacts with Ste12 and Tec1. Earlier it has been noted that Flo8 and Mss11 bind indirectly to Uas2-1 of the Sta1 promoter by interacting with Ste12 and Tec1 [52]. Although the interactions are not specified in the filamentation process, proteins such as Flo8, Mss10/Msn1, Mss11, Ste12, and Tec1, are known to activate the transcription of Sta1 in the absence of glucose (starvation). Moreover, Fus1 and Spa2 were observed to contribute to Bni1 localization during pheromone response, although they were not shown on the KEGG database [53]. Las17 is known to be active in response to osmotic stress. Here, Las17 interacts with Bem1 and Sho1 through SH3 binding sites [54]. It is know that Las17 has a high number of SH3 binding partners. However, the significance of these results remains to be determined.

**Figure 6:**
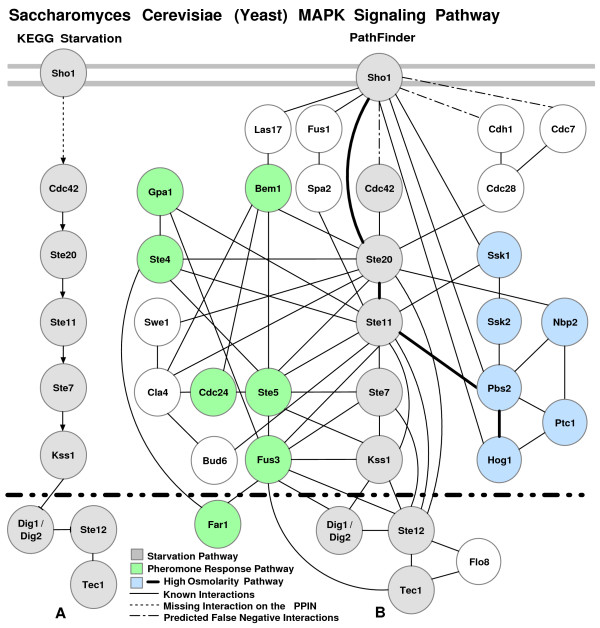
**The filamentation signaling pathway recovered completely**. (A) The main chain of the filamentation pathway (KEGG Database), (B) PathFinder output for Sho1-Tec1 pair (100% recall, 12% precision). For (A), the dashed links indicate interactions that do not exists in the database. For (B), the interactions that were predicted as false negatives were shown with dashed lines. The proteins that were not on the main chain of any pathway were not colored, whereas proteins that are part of other pathways were colored.

The missing pathway protein, Cdc42, shown in Figure 5B is due to a missing interaction in the PPIN. To recover this false negative interaction edge, we incorporated inferred links into our network (See *Recovering false negative interactions*) and acquired the pathway segment in Figure 6B.

In this signaling pathway segment, all interacting proteins of the pathway and their interactions were recovered. The inferred links were marked with dotted lines. Since, the number of edges in the PPIN were increased, the number of proteins and possible links were also greater in number in the final pathway segment. We improved the recall rate (75% to 100%), whereas the precision declined slightly (17% to 12%). However, most of the additions were functionally related proteins and as seen in Figure 1, MAPK pathways share proteins. For instance, we have recovered the majority of the HOG MAPK pathway, which is a segment starting from Sho1 and ending at Hog1 (shown with bold lines in Figure 6B).

Here we have not only shown that our methodology can recover missing links on this pathway with additional empirical information, but also how functional properties are utilized to bring pathway segments together. As noted earlier [11], signaling pathways are not only individual paths but also inter connected functional chains.

## Conclusion

In this paper, we proposed the PathFinder system to find possible pathway segments. Biological annotations are used to represent proteins. Association rules are employed to capture the characteristics of known signaling pathways. We filtered the protein-protein interaction network by integrating other biological knowledge into the network. During a user's query, the paths in the protein-protein interaction network are retrieved and validated against the acquired association rules. False negative interactions on these paths are identified using protein family information. We demonstrate the advantages of our methodology via the yeast protein-protein interaction network.

## Competing interests

The author(s) declares that there are no competing interests.

## Authors' contributions

GB completed the study and wrote the manuscript. JY supervised the project and revised the manuscript.

All authors read and approved the final version of the manuscript.

## References

[B1] Fields S, Song O (1989). A novel genetic system to detect protein-protein interactions. Nature.

[B2] Uetz P, Giot L, Cagney G, Mansfield TA, Judson RS, Knight JR, Lockshon D, Narayan V, Srinivasan M, Pochart P, Qureshi-Emili A, Li Y, Godwin B, Conover D, Kalbfleisch T, Vijayadamodar G, Yang M, Johnston M, Fields S, Rothberg JM (2000). A comprehensive analysis of protein-protein interactions in Saccharomyces cerevisiae. Nature.

[B3] Ito T, Chiba T, Ozawa R, Yoshida M, Hattori M, Sakaki Y (2001). A comprehensive two-hybrid analysis to explore the yeast protein interactome. Proc Natl Acad Sci USA.

[B4] Reboul J, Vaglio P, Rual JF, Lamesch P, Martinez M, Armstrong CM, Li S, Jacotot L, Bertin N, Janky R, Moore T, Hudson JR, Hartley JL, Brasch MA, Vandenhaute J, Boulton S, Endress GA, Jenna S, Chevet E, Papasotiropoulos V, Tolias PP, Ptacek J, Snyder M, Huang R, Chance MR, Lee H, Doucette-Stamm L, Hill DE, Vidal M (2003). C. elegans ORFeome version 1.1: experimental verification of the genome annotation and resource for proteome-scale protein expression. Nat Genet.

[B5] Giot L (2003). A protein interaction map of Drosophila melanogaster. Science.

[B6] Li S, Armstrong CM, Bertin N, Ge H, Milstein S, Boxem M, Vidalain PO, Han JD, Chesneau A, Hao T, Goldberg DS, Li N, Martinez M, Rual JF, Lamesch P, Xu L, Tewari M, Wong SL, Zhang LV, Berriz GF, Jacotot L, Vaglio P, Reboul J, Hirozane-Kishikawa T, Li Q, Gabel HW, Elewa A, Baumgartner B, Rose DJ, Yu H, Bosak S, Sequerra R, Fraser A, Mango SE, Saxton WM, Strome S, Van Den Heuvel S, Piano F, Vandenhaute J, Sardet C, Gerstein M, Doucette-Stamm L, Gunsalus KC, Harper JW, Cusick ME, Roth FP, Hill DE, Vidal M (2004). A map of the interactome network of the metazoan C. elegans. Science.

[B7] Gavin AC (2002). Functional organization of the yeast proteome by systematic analysis of protein complexes. Nature.

[B8] Ho Y (2002). Systematic identification of protein complexes in Saccharomyces cerevisiae by mass spectrometry. Nature.

[B9] Alberts B, Johnson A, Lewis J, Raff M, Roberts K, Walter P (2002). Molecular Biology of the Cell [Book and CD-ROM]. Garland Science.

[B10] Bateson W Mendel's Principles of Heredity. Genetics Heritage Pr 1909.

[B11] Neves SR, Iyengar R (2002). Modeling of signaling networks. Bioessays.

[B12] Choi C, Crass T, Kel A, Kel-Margoulis O, Krull M, Pistor S, Potapov A, Voss N, Wingender E (2004). Consistent re-modeling of signaling pathways and its implementation in the TRANSPATH database. Genome Inform Ser.

[B13] Sachs K, Perez O, Pe'er D, Lauffenburger DA, Nolan GP (2005). Causal Protein-Signaling Networks Derived from Multiparameter Single-Cell Data. Science.

[B14] Graeber TG, Eisenberg D (2001). Bioinformatic identification of potential autocrine signaling loops in cancers from gene expression profiles. Nat Genet.

[B15] Ideker T, Thorsson V, Ranish JA, Christmas R, Buhler J, Eng JK, Bumgarner R, Goodlett DR, Aebersold R, Hood L (2001). Integrated genomic and proteomic analyses of a systematically perturbed metabolic network. Science.

[B16] Tucker CL, Gera JF, Uetz P (2001). Towards an understanding of complex protein networks. Trends in Cell Biology.

[B17] Zien A, Küffner R, Zimmer R, Lengauer T, Altman R, et al (2000). Analysis of Gene Expression Data with Pathway Scores. ISMB00.

[B18] Stefen M, Petti A, Aach J, D'haeseleer P, Church G (2002). Automated modelling of signal transduction networks. BMC Bioinformatics.

[B19] Liu Y, Zhao H (2004). A computational approach for ordering signal transduction pathway components from genomics and proteomics Data. BMC Bioinformatics.

[B20] Scott J, Ideker T, Karp RM, Sharan R (2006). Efficient algorithms for detecting signaling pathways in protein interaction networks. J Comput Biol.

[B21] Shlomi T, Segal D, Ruppin E, Sharan R (2006). QPath: a method for querying pathways in a protein-protein interaction network. BMC Bioinformatics.

[B22] Agrawal R, Imielinski T, Swami AN, Buneman P, Jajodia S (1993). Mining Association Rules between Sets of Items in Large Databases. Proc of the 1993 ACM SIGMOD Int Conference on Management of Data.

[B23] Walhout AJ, Boulton SJ, Vidal M (2000). Yeast two-hybrid systems and protein interaction mapping projects for yeast and worm. Yeast.

[B24] Walhout AJ, Sordella R, Lu X, Hartley JL, Temple GF, Brasch MA, Thierry-Mieg N, Vidal M (2000). Protein Interaction Mapping in C. elegans Using Proteins Involved in Vulval Development. Science.

[B25] Edwards AM, Kus B, Jansen R, Greenbaum D, Greenblatt J, Gerstein M (2002). Bridging structural biology and genomics: assessing protein interaction data with known complexes. Trends Genet.

[B26] von Mering C, Krause R, Snel B, Cornell M, Oliver SG, Fields S, Bork P (2002). Comparative assessment of large-scale data sets of protein-protein interactions. Nature.

[B27] Grigoriev A (2003). On the number of protein-protein interactions in the yeast proteome. Nuc Ac Res.

[B28] Ito T, Ota K, Kubota H, Yamaguchi Y, Chiba T, Sakuraba K, Yoshida M (2002). Roles for the Two-hybrid System in Exploration of the Yeast Protein Interactome. Mol Cell Proteomics.

[B29] Ashburner M, Ball CA, Blake JA, Botstein D, Butler H, Cherry JM, Davis AP, Dolinski K, Dwight SS, Eppig JT, Harris MA, Hill DP, Issel-Tarver L, Kasarskis A, Lewis S, Matese JC, Richardson JE, Ringwald M, Rubin GM, Sherlock G (2000). Gene ontology: tool for the unification of biology. The Gene Ontology Consortium. Nat Genet.

[B30] Kanehisa M, Goto S (2000). KEGG: Kyoto Encyclopedia of Genes and Genomes. Nuc Ac Res.

[B31] Mewes HW, Heumann K, Kaps A, Mayer K, Pfeiffer F, Stocker S, Frishman D (1999). MIPS: a database for genomes and protein sequences. Nuc Ac Res.

[B32] Campagne F, Neves S, Chang CW, Skrabanek L, Ram PT, Iyengar R, Weinstein H (2004). Quantitative information management for the biochemical computation of cellular networks. Sci STKE.

[B33] Gough NR, Adler EM, Ray LB (2004). Focus Issue: Cell Signaling-Making New Connections. Sci STKE.

[B34] Ruepp A, Zollner A, Maier D, Albermann K, Hani J, Mokrejs M, Tetko I, Guldener U, Mannhaupt G, Munsterkotter M, Mewes HW (2004). The FunCat, a functional annotation scheme for systematic classification of proteins from whole genomes. Nuc Ac Res.

[B35] Brin S, Motwani R, Silverstein C, Peckham J (1997). Beyond Market Baskets: Generalizing Association Rules to Correlations. SIGMOD Proceedings ACM SIGMOD International Conference on Management of Data, May 13–15, 1997, Tucson, Arizona, USA.

[B36] Bebek G (2007). Analyzing and Modeling Large Biological Networks: Inferring Signal Transduction Pathways. PhD thesis.

[B37] Deane CM, Salwinski L, Xenarios I, Eisenberg D (2002). Protein interactions: two methods for assessment of the reliability of high throughput observations. Mol Cell Proteomics.

[B38] Deng M, Sun F, Chen T (2003). Assessment of the reliability of protein-protein interactions and protein function prediction. Pac Symp Biocomput.

[B39] Bader JS, Chaudhuri A, Rothberg JM, Chant J (2004). Gaining confidence in high-throughput protein interaction networks. Nat Biotechnol.

[B40] Sharan R, Suthram S, Kelley RM, Kuhn T, McCuine S, Uetz P, Sittler T, Karp RM, Ideker T (2005). Conserved patterns of protein interaction in multiple species. Proc Natl Acad Sci USA.

[B41] Qi Y, Klein-Seetharaman J, Bar-Joseph Z (2005). Random forest similarity for protein-protein interaction prediction from multiple sources. Pac Symp Biocomput.

[B42] Grigoriev A (2001). A relationship between gene expression and protein interactions on the proteome scale: analysis of the bacteriophage T7 and the yeast Saccharomyces cerevisiae. Nuc Ac Res.

[B43] Suthram S, Shlomi T, Ruppin E, Sharan R, Ideker T (2006). A direct comparison of protein interaction confidence assignment schemes. BMC Bioinformatics.

[B44] Huh WK, Falvo JV, Gerke LC, Carroll AS, Howson RW, Weissman JS, O'Shea EK (2003). Global analysis of protein localization in budding yeast. Nature.

[B45] Stark C, Breitkreutz BJ, Reguly T, Boucher L, Breitkreutz A, Tyers M (2006). BioGRID: a general repository for interaction datasets. Nuc Ac Res.

[B46] Ball CA, Awad IAB, Demeter J, Gollub J, Hebert JM, Hernandez-Boussard T, Jin H, Matese JC, Nitzberg M, Wymore F, Zachariah ZK, Brown PO, Sherlock G (2005). The Stanford Microarray Database accommodates additional microarray platforms and data formats. Nuc Ac Res.

[B47] Bebek G, Berenbrink P, Cooper C, Friedetzky T, Nadeau J, Sahinalp SC (2007). Improved Duplication Models for Proteome Network Evolution. RECOMB 2005 Ws on Regulatory Genomics.

[B48] Bateman A, Coin L, Durbin R, Finn RD, Hollich V, Griffths-Jones S, Khanna A, Marshall M, Moxon S, Sonnhammer ELL, Studholme DJ, Yeats C, Eddy SR (2004). The Pfam protein families database. Nuc Ac Res.

[B49] Madhani HD, Styles CA, Fink GR (1997). MAP kinases with distinct inhibitory functions impart signaling specificity during yeast differentiation. Cell.

[B50] Dohlman HG, Song J, Ma D, Courchesne WE, Thorner J (1996). Sst2, a negative regulator of pheromone signaling in the yeast Saccharomyces cerevisiae: expression, localization, and genetic interaction and physical association with Gpa1. Mol Cell Biol.

[B51] Konopka JB, Jenness DD (1991). Genetic fine-structural analysis of the Saccharomyces cerevisiae alpha-pheromone receptor. Cell Regul.

[B52] Kim TS, Kim HY, Yoon JH, Kang HS (2004). Recruitment of the Swi/Snf Complex by Ste12-Tec1 Promotes Flo8-Mss11-Mediated Activation of STA1 Expression. Mol Cell Biol.

[B53] Nelson B, Parsons AB, Evangelista M, Schaefer K, Kennedy K, Ritchie S, Petryshen TL, Boone C (2004). Fus1p Interacts With Components of the Hog1p Mitogen-Activated Protein Kinase and Cdc42p Morphogenesis Signaling Pathways to Control Cell Fusion During Yeast Mating. Genetics.

[B54] Winters MJ, Pryciak PM (2005). Interaction with the SH3 domain protein Bem1 regulates signaling by the Saccharomyces cerevisiae p21-activated kinase Ste20. Mol Cell Biol.

